# Pooling RT-qPCR testing for SARS-CoV-2 in 1000 individuals of healthy and infection-suspected patients

**DOI:** 10.1038/s41598-020-76043-z

**Published:** 2020-11-03

**Authors:** Yosuke Hirotsu, Makoto Maejima, Masahiro Shibusawa, Yuki Nagakubo, Kazuhiro Hosaka, Kenji Amemiya, Hitomi Sueki, Miyoko Hayakawa, Hitoshi Mochizuki, Toshiharu Tsutsui, Yumiko Kakizaki, Yoshihiro Miyashita, Masao Omata

**Affiliations:** 1 Genome Analysis Center, Yamanashi Central Hospital, 1-1-1 Fujimi, Kofu, Yamanashi Japan; 2 Division of Microbiology in Clinical Laboratory, Yamanashi Central Hospital, 1-1-1 Fujimi, Kofu, Yamanashi Japan; 3 Division of Genetics and Clinical Laboratory, Yamanashi Central Hospital, 1-1-1 Fujimi, Kofu, Yamanashi Japan; 4 Department of Gastroenterology, Yamanashi Central Hospital, 1-1-1 Fujimi, Kofu, Yamanashi Japan; 5 Lung Cancer and Respiratory Disease Center, Yamanashi Central Hospital, 1-1-1 Fujimi, Kofu, Yamanashi Japan; 6 The University of Tokyo, 7-3-1 Hongo, Bunkyo-ku, Tokyo, Japan

**Keywords:** Viral infection, Epidemiology, Genetics research

## Abstract

Severe acute respiratory coronavirus 2 (SARS-CoV-2) testing reagents are expected to become scarce worldwide. However, little is known regarding whether pooling of samples accurately detects SARS-CoV-2. To validate the feasibility of pooling samples, serial dilution analysis and spike-in experiments were conducted using synthetic DNA and nucleic acids extracted from SARS-CoV-2-positive and -negative patients. Furthermore, we studied 1000 individuals, 667 of whom were “healthy” individuals (195 healthcare workers and 472 hospitalized patients with disorders other than COVID-19 infection), and 333 infection-suspected patients with cough and fever. Serial dilution analysis showed a limit of detection of around 10–100 viral genome copies according to the protocol of the National Institute of Infectious Diseases, Japan. Spike-in experiments demonstrated that RT-qPCR detected positive signals in pooled samples with SARS-CoV-2-negative and -positive patients at 5-, 10-, 20-fold dilutions. By screening with this pooling strategy, by the end of April 2020 there were 12 SARS-CoV-2-positive patients in 333 infection-suspected patients (3.6%) and zero in 667 “healthy” controls. We obtained these results with a total of 538 runs using the pooling strategy, compared with 1000 standard runs. In a prospective study, we successfully detected SARS-CoV-2 using 10- to 20-fold diluted samples of nasopharyngeal swabs from eighteen COVID-19 patients with wide ranges of viral load. Pooling sample is feasible for conserving test reagents and detecting SARS-CoV-2 in clinical settings. This strategy will help us to research the prevalence infected individuals and provide infected-status information to prevent the spread of the virus and nosocomial transmission.

## Introduction

A new emergent coronavirus, severe acute respiratory syndrome coronavirus 2 (SARS-CoV-2), broke out in late 2019 in Wuhan, China, spreading across the world within a few months^1,2^. SARS-CoV-2 infection has resulted in a high number of patients presenting with coronavirus disease 2019 (COVID-19) and a mortality rate of 2.3% in China and 7.2% in Italy^3^. SARS-CoV-2 has been spread by infected people with mild or no symptoms as well as severe symptoms^4^. The World Health Organization raised a global warning, declaring a pandemic, and announced the need for a testing system for patients with suspected SARS-CoV-2 infection. The development of an efficient and accurate testing system for SARS-CoV-2 is an urgent issue.

The explosive increase in COVID-19 cases has depleted medical resources required to protect against infection^5^. For instance, face shields, infection control gowns, N95 masks, surgical masks, and personal protective equipment are in short supply in hospitals^6^. As a result, the risk of infection among medical staff and resultant nosocomial infection is expected to be high. To prevent viral spread, careful cleaning in and around the hospital room is performed^7^. In addition, screening tests for healthcare workers, ambulatory patients, hospitalized patients and asymptomatic cases are important for early detection of SARS-CoV-2 and prevention of nosocomial infections. Prophylactic testing can help protect healthcare workers and patients in clinical settings.

As SARS-CoV-2 has spread, tens of thousands of tests have been performed daily worldwide. Quantitative reverse transcription-PCR (RT-qPCR) is now widely used for the detection of SARS-CoV-2^8^. RT-qPCR is conducted by performing viral RNA extraction, reverse transcription of extracted RNA, and RT-qPCR detection with primers and a fluorescent probe. It is expected that the supply of reagents will be depleted with growing global demand for SARS-CoV-2 testing. In particular, virus nucleic acid extraction reagents and enzyme-containing reagents for RT-qPCR are required to conduct testing. In the U.S., testing has been stopped in some areas due to lack of reagents^9^. Therefore, it is important to consider how to efficiently conduct RT-qPCR testing with accuracy while conserving reagents.

Analysis of pooled samples is a time- and cost-saving method for diagnosis of infectious diseases^10^. Robert et al. successfully identified syphilis from pooled samples in 1943^11^. Pooling strategies have also been used for the detection of other pathogens including hepatitis B and C viruses, human immunodeficiency virus, *Chlamydia trachomatis* and *Neisseria gonorrhoeae*^12–16^. A pooling strategy was previously conducted for screening for SARS-CoV-2 in the U.S.^9,17^. In this study, we validated the pooling strategy for detecting SARS-CoV-2 using multiple nasopharyngeal swabs. This method would help save time and reagents in short supply and prevent delays in reporting results. This could provide prevalence data for the ongoing infection status in our district, west of Tokyo, Japan.

## Results

### Serial dilution assay using plasmid control and SARS-CoV-2 negative individuals

RT-qPCR for SARS-CoV-2 was conducted based on protocols developed by the National Institute of Infectious Diseases (NIID) in Japan^18^. This assay targets two sites of the nucleocapsid (*N*) gene of SARS-CoV-2. We previously conducted serial dilution experiments with a positive control plasmid and observed that primer/probe targeting of the N2 site was more sensitive than that of the N1 site^19^.

**Figure 1:**
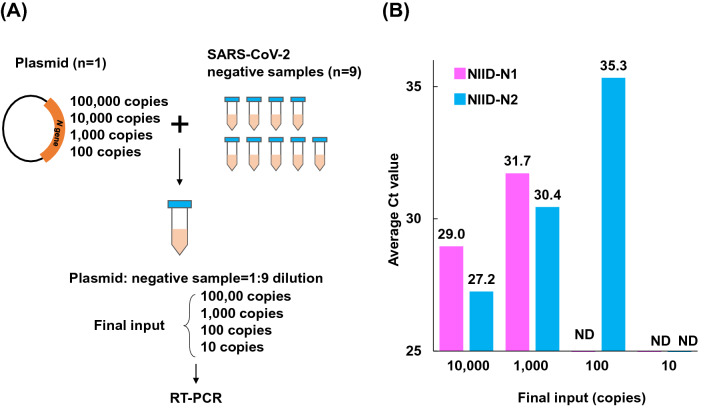
Serial dilution analysis with synthetic plasmids and nasopharyngeal swabs from SARS-CoV-2 negative individuals. (**A**) Scheme of preparing serial dilution solutions. The synthetic plasmid containing the *N* gene of SARS-CoV-2 were diluted to concentrations of 100,000, 10,000, 1000 and 100 copies. These plasmid solutions were then tenfold diluted with nucleic acids extracted from nasopharyngeal swabs from SARS-CoV-2 negative individuals (n = 9). RT-qPCR analysis had already validated that no amplification was observed in these negative patients in advance. RT-qPCR analyses were conducted using serial dilution solution with final input copy numbers (range 10–10,000). (**B**) Average threshold cycle (Ct) was determined by RT-qPCR. Two sets of primers and probes (pink, NIID-N1; blue, NIID-N2) were used according to the NIID, Japan, protocol. The experiment was conducted three times in duplicate.

To assess the feasibility of pooling samples, we diluted the plasmid control containing the *N* gene of SARS-CoV-2 with total nucleic acids extracted from SARS-CoV-2-negative individuals. First, we diluted the synthetic plasmid control to different concentrations (100,000, 10,000, 1000 and 100 copies). These different diluted samples were mixed with nucleic acids extracted from nasopharyngeal swabs of SARS-CoV-2-negative individuals at a ratio of 1:9 ratio. As a result, the expected input amounts of 10,000, 1000, 100 and 10 copies were analyzed by RT-qPCR (Fig. 1A). The results showed primer/probe targeting of the N1 site detected 10,000 and 1000 copies of plasmid but did not detect 100 and 10 copies; whereas the N2 site detected at 100 copies (Fig. 1B). These results suggested the limits of detection were 100–1000 copies and 10–100 copies for the N1 and N2 sites, respectively.

### Spike-in assay using SARS-CoV-2 positive and negative samples

**Figure 2:**
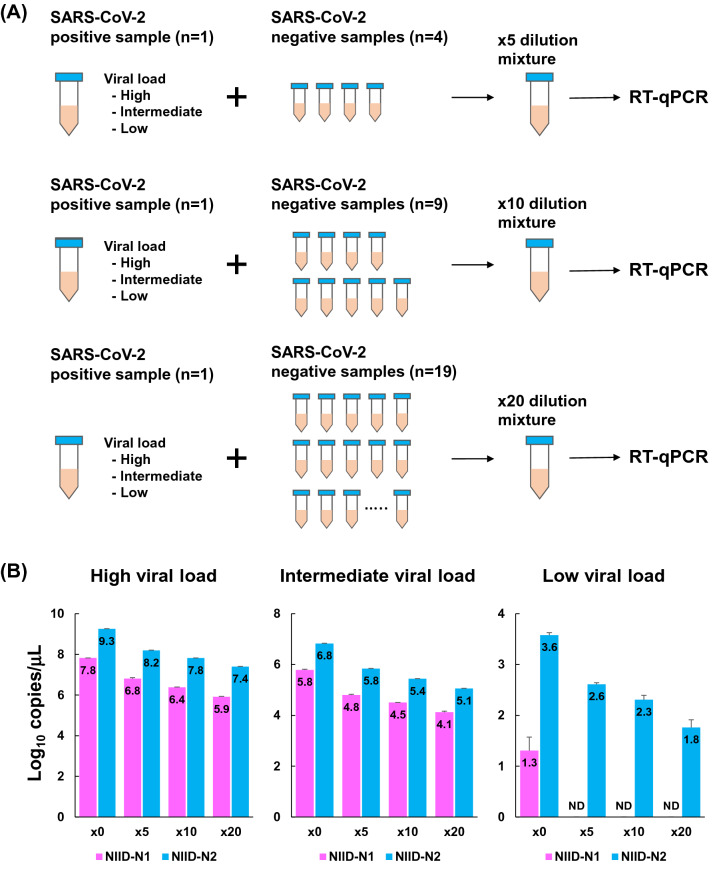
Spike-in assay using SARS-CoV-2-positive and -negative nasopharyngeal swabs from patients. (**A**) Scheme of preparing spike-in solutions. SARS-CoV-2-positive samples with high, intermediate and low viral copies were used. These different viral loads were diluted with 4, 9 and 19 SARS-CoV-2-negative samples. The final solution was made at × 5, × 10 and × 20 dilutions and used for RT-qPCR analysis. (**B**) RT-qPCR analysis determined the copy numbers in the spike-in solutions. Each of the three patients had a different viral load corresponding to low, intermediate, and high. These SARS-CoV-2 positive samples were diluted with negative samples. The assay was used with NIID-N1 (pink) and NIID-N2 (blue). Bar plot shows the copy number (log_10_ copies/μL) in original (× 0) and diluted (× 5, × 10 and × 20) samples. All data represent the mean ± SD.

To test whether this pooling method could be applied in clinical practice, we mixed samples from three confirmed SARS-CoV-2-positive patients with varying virus loads and negative individuals. Each of these three patients had a different viral load corresponding to low, intermediate, and high. Samples from the three patients were diluted in negative samples obtained from healthy individuals at a ratio of 1:4, 1:9, and 1:19 (Fig. 2A). As a result, samples were prepared at 5-, 10- and 20-fold dilution, respectively.

These samples were subjected to RT-qPCR. As a result, the original viral load in the undiluted sample was calculated to be 7.8 log_10_ (high viral load), 5.8 log_10_ (intermediate), and 1.3 log_10_ (low) at the N1 site, and 9.3 log_10_ (high), 6.8 log_10_ (intermediate), and 3.6 log_10_ (low) at the N2 site (Fig. 2B).

The primer/probe targeting the N1 site detected nucleic acids from high and moderate viral load samples, but not from the low viral load sample when samples were diluted (Fig. 2B). In contrast, the N2 site assay detected viral nucleic acids from high, moderate and low viral load samples (Fig. 2B). These results again showed N2 is a superior target for analyzing the pooled samples compared with the N1 site.

### SARS-CoV-2 test for suspected COVID-19 patients, healthcare workers and hospitalized patients

**Figure 3:**
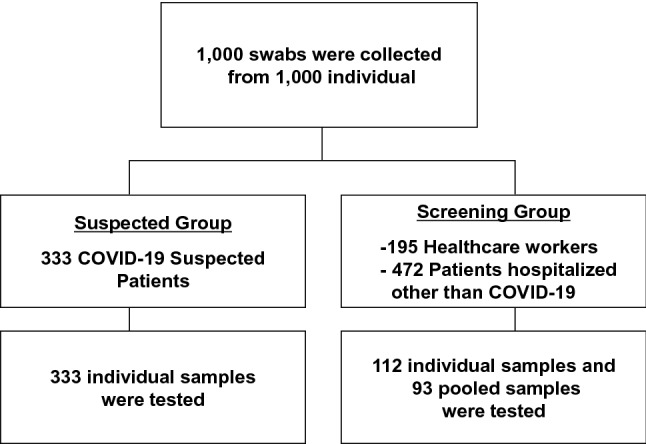
Flow diagram for testing of 1000 individuals. The flow diagram illustrates the 1000 individuals who were divided into suspected or screening groups. The suspected group included 333 suspected COVID-19 patients. The screening group included 195 healthcare workers and 472 patients hospitalized for conditions other than COVID-19.

From March 11 to April 28, 2020, we studied a total of 1000 individuals including 333 suspected COVID-19 patients, 472 hospitalized patients with disorders other than COVID-19 and 195 healthcare workers (Fig. 3, Table 1). Of 1000 individuals, 445 were tested individually (Fig. 3). In the remaining 555 individuals, we made 93 pools (average mixed 5.97 swabs/pool, range 5–10/pool) to be tested.

**Table 1:** Prevalence of SARS-CoV-2 in infection-suspected patients and screening group.

	Suspected group	Screening group		Total
	COVID-19 suspected patients^a^	Healthcare workers	Patients hospitalized other than COVID-19	
**Number of patients**	333	195	472	1000
COVID-19 positive/negative (%)	12 (3.6%)/321 (97.7%)	0 (0%)/195 (100%)	0 (0%)/472(100%)	12 (1.2%)/988 (98.8%)
**Total number of tested samples**	333	91	114	538
Individual	333	65	47	445
Pooled	0	26	67	93^b^

^a^This group includes the 12 COVID-19-confirmed patients.

^b^Ninety-three batched samples (pools of five to 10 swabs from 555 individuals).

RT-qPCR showed the prevalence of COVID-19 was 3.6% (12/333) of infection-suspected patients, whereas no positivity was detected in either healthcare workers or hospitalized patients in our district (Table 1). To see if we missed any cases with very low virus loads and individuals in the incubation period of the infection, we followed the conditions of these patients. To date, we have prospectively followed up the healthcare workers and COVID-19-negative hospitalized individuals and have not observed any apparent symptoms within the average hospital stay of 10–12 days duration, suggesting nosocomial infections could be prevented in our hospital.

### Pooling strategy detected SARS-CoV-2 positive samples with a wide range of viral load

**Figure 4:**
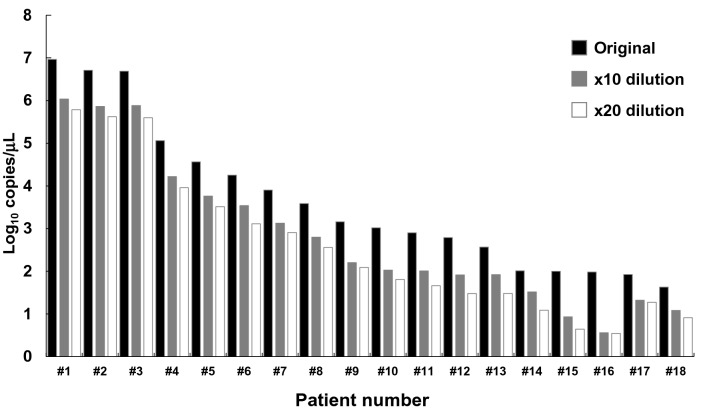
Pooling strategy detected SARS-CoV-2 in samples with a wide range of viral load. We diluted SARS-CoV-2 positive samples with negative samples. The original sample (black box), × 10 diluted (gray box) and × 20 diluted (white box) samples were tested by RT-qPCR. All samples were collected from 18 patients (#1–18) with high to low viral load.

We prospectively collected nasopharyngeal swabs from 18 patients infected with SARS-CoV-2. These samples harbored a wide range of viral load (mean = 3.6 log_10_ copies, range: 1.6–7.0 log_10_ copies). We mixed SARS-CoV-2 positive and negative samples at a ratio of 1:9 (tenfold dilution) and 1:19 (20-fold dilution). A total of 54 samples including original and diluted samples were assessed by RT-qPCR with primer/probes in N2 site. From high (#1) to low viral load (#18) of original samples, we detected the positive signal in 10- and 20-fold dilutions (Fig. 4). These results demonstrated the pooling strategy was feasible for detecting COVID-19 patients in routine clinical practice.

## Discussion

Here, we show the utility of a pooling strategy for detecting SARS-CoV-2. Serial dilution analysis with control plasmid DNA and nucleic acids extracted from healthy individuals showed that the limit of detection was estimated at 10–100 copies using the N2 site, according to the NIID protocol. The spike-in analysis with positive and negative SARS-CoV-2 samples demonstrated that SARS-CoV-2 was detectable by RT-qPCR even in pooled samples with low viral loads (Fig. 2B). Actually, SARS-CoV-2 RNA was detected in clinical samples by pooling of 10 and 20 individual samples in one batch (Fig. 4). In addition, we showed the applicability of the pooling strategy for screening large cohorts of healthcare workers and patients in hospitals (Table 1).

If the PCR efficiency is 100%, pooling of five samples theoretically decreases the viral loads from the original value to 0.7 log_10_ (Ct value increases by 2.3), 10 samples decreases to 1.0 log_10_ (Ct increases by 3.3), and 20 samples decreases to 1.3 log_10_ (Ct increase by 4.3). In the case of the N2 site of the NIID assay, the limit of detection was estimated around 10–100 viral copies from the serial dilution assay. Because primer and probe set of N2 site was superior to that of N1 site (Fig. 2B), pooling strategy could be applied using N2 site.

Our data suggests pooling of five, 10 and 20 samples as one batch is feasible when the original viral load is 50–500, 100–1000 and 200–2000 copies, respectively. When positive results in a pooled sample are observed, the samples in these pools must then be retested individually. This strategy is effective for conservation of reagents and reducing time for reporting of SARS-CoV-2 testing. In fact, in the U.S. where more PCR assays are clearly needed, 292 pools of approximately 10 samples per pool were made from 2888 individual samples, and the reported positivity rate was 0.07% (2/2888)^17^. However, the sensitivity of this test was not investigated.

The viral load reaches a peak around the time of symptom onset and then gradually declines in COVID-19 patients^20^. The median initial viral load has been reported as 6.17 log_10_ copies (range 4.18–7.13) in severe cases and 5.11 log_10_ copies (range 3.91–7.56) in mild cases^21^. These observed viral loads in COVID-19 are within the detectable limits of pooled samples. It is possible that totally asymptomatic COVID-19-positive individuals may exist and could have high viral loads^21^, therefore screening of asymptomatic individuals by PCR testing could be effective on prevention of viral spreading^22^. This pooling method would become a powerful tool to detect asymptomatic “super spreaders” with heavy viral loads. Such super spreaders are key players for the increasing infection trend. Recently, a short report on apparently healthy individuals from University Hospital, Tokyo in Japan, reported a 7.4% (5/67) prevalence of PCR-confirmed SARS-CoV-2 positivity on April 21, 2020^23^. Fortunately, our district (100–150 km from Tokyo) been not been obviously invaded by this virus as of the end of April, 2020.

A limitation of this study is a loss of sensitivity, as we have shown, of approximately 1.3–1.5 log_10_ when 10 samples were pooled. In two to three weeks of follow-up, however, none of the screened nor suspected group developed COVID-19 symptoms at the time of manuscript preparation at the end of April 2020.

Collectively, this pooling strategy may help to detect early infections and prevent nosocomial transmission in hospitals. Screening of inpatients, emergency admissions, physicians and health care providers, as in this study, can be useful for infection control. This method may help to elucidate the future trend of the infection and reveal the prevalence of COVID-19 patients without losing any accuracy despite fewer tested samples.

## Methods

### Patients and medical staff samples

We collected nasopharyngeal swabs between March 11 and April 28, 2020 at Yamanashi Central Hospital, Japan. All samples were obtained with cotton swabs and universal transport media (Copan, Murrieta, CA). To screen whether medical staff and hospitalized patients were infected with SARS-CoV-2, we tested a total of 1000 samples from 1000 individuals including 333 COVID-19-suspected patients, 195 healthcare workers and 472 patients hospitalized for conditions other than COVID-19. Suspected COVID-19 patients (n = 333) had symptoms including fever, fatigue, cough and/or respiratory failure. Using the pooling strategy, we tested a total of 538 samples (445 individually and 93 pooled) from 1000 individuals. One pooled batch comprised 5 to 10 samples.

The Institutional Review Board of clinical research and genome research committee at Yamanashi Central Hospital approved this retrospective study (Approval No. C2019-30) and the use of an opt-out consent method. Consent will be obtained after disclosing information about this research. We ensured patients have the opportunity to refuse to participate in the research. Patients will not be disadvantaged if patients refuse to participation. The requirement for written informed consent was waived by clinical research and genome research committee at Yamanashi Central Hospital. All methods were carried out in accordance with relevant guidelines and regulations.

### Viral nucleic acid extraction

Total nucleic acid was automatically isolated from nasopharyngeal swabs using the MagMax Viral/Pathogen Nucleic Acid Isolation Kit (Thermo Fisher Scientific, Waltham, MA) on an automated machine (KingFisher Duo Prime, Thermo Fisher) according to the manufacturer’s protocol. Briefly, we added 400 µL of viral transport media, 10 µL of proteinase K, 530 μL binding solution, 20 μL total nucleic acid binding beads, 1 mL wash buffer, and 1 mL or 0.5 mL of 80% ethanol to each well of a deep-well 96-well plate. One hundred microliters of elution solution was added to the elution strip. Total nucleic acids were stored at − 80 °C until required for further RT-qPCR analysis.

### One-step RT-qPCR

The protocol was designed by the NIID, Japan. To detect SARS-CoV-2 (NCBI Reference Sequence: NC_045512.2), we performed one-step RT-qPCR according to the NIID protocol with minor modifications (version 2.7)^18^. The primer/probe set tests two sites (N1 and N2) of the *N* gene of SARS-CoV-2^18,19^.

For N1 detection with the NIID assay, the reaction mixture comprised 5 μL of 4 × TaqMan Fast Virus 1-Step Master Mix, 1.2 μL of 10 μM forward primer (5′-CACATTGGCACCCGCAATC-3′), 1.6 μL of 10 μM reverse primer (5′-GAGGAACGAGAAGAGGCTTG-3′), 0.8 μL of 5 μM probe (5′-FAM-ACTTCCTCAAGGAACAACATTGCCA-TAMRA-3′), 6.4 μL of nuclease-free water (Thermo Fisher Scientific), and 5 μL of sample in a 20 μL total volume. For N2 detection, the reaction mixture comprised 5 μL of 4 × TaqMan Fast Virus 1-Step Master Mix, 1.0 μL of 10 μM forward primer (5′-AAATTTTGGGGACCAGGAAC-3′), 1.4 μL of 10 μM reverse primer (5′-TGGCAGCTGTGTAGGTCAAC-3′), 0.8 μL of 5 μM probe (5′-FAM-ATGTCGCGCATTGGCATGGA-TAMRA-3′), 6.8 μL of nuclease-free water, and 5 μL of sample in a 20 μL total volume. The expected amplicon sizes for the NIID-N1 and N2 are 128 bp and 158 bp, respectively. For the internal positive control, the human ribonuclease P 30 subunit (*RPP30*) gene was used^19^.

The RT-qPCR assays were conducted on a StepOnePlus Real-Time PCR Systems machine (Thermo Fisher Scientific) with the following cycling conditions: 50 °C for 5 min for reverse transcription, 95 °C for 20 s, and 45 cycles of 95 °C for 3 s and 60 °C for 30 s^24,25^. The threshold line was set at 0.2. The threshold cycle (Ct) value was assigned to each PCR reaction and the amplification curve was visually assessed. The absolute copy number of viral loads was determined using serial diluted DNA control targeting the *N* gene of SARS-CoV-2 (Integrated DNA Technologies, Coralville, IA).

### Serial dilution assay using SARS-CoV-2 DNA plasmid control

We purchased the SARS-CoV-2 DNA plasmid control (Integrated DNA Technologies, catalog #10006625), which consists of 200,000 copies/µL containing the complete *N* gene^19^. We prepared a serial dilution of the plasmid control (100,000, 10,000, 1000 and 100 copies) using nuclease-free water (Thermo Fisher Scientific) to assess the limit of detection. Serial diluted plasmid was mixed with total nucleic acid extracted from SARS-CoV-2-negative individuals at a ratio of 1:9. Plasmid controls with a tenfold dilution of the final concentration were analyzed by RT-qPCR.

### Spike-in assay using COVID-19-infected and non-infected patients

Representative nucleic acid samples extracted from three COVID-19 patients were used for spike-in analysis. These samples contained high, intermediate and low virus loads. Using these three samples, we examined to what extent SARS-CoV-2 could be detected when multiple samples were pooled. SARS-CoV-2-positive and -negative samples were mixed in ratios of 1:4, 1:9, and 1:19, creating pooled samples of 5-, 10- and 20-fold dilutions, respectively.

## Data Availability

All data generated or analysed during this study are included in this published article.
